# A Sequence Type 23 Hypervirulent *Klebsiella pneumoniae* Strain Presenting Carbapenem Resistance by Acquiring an IncP1 *bla*_KPC-2_ Plasmid

**DOI:** 10.3389/fcimb.2021.641830

**Published:** 2021-06-01

**Authors:** Rushuang Yan, Ye Lu, Yiwei Zhu, Peng Lan, Shengnan Jiang, Jun Lu, Ping Shen, Yunsong Yu, Jiancang Zhou, Yan Jiang

**Affiliations:** ^1^ Department of Infectious Diseases, Sir Run Run Shaw Hospital, Zhejiang University School of Medicine, Hangzhou, China; ^2^ Key Laboratory of Microbial Technology and Bioinformatics of Zhejiang Province, Hangzhou, China; ^3^ Regional Medical Center for National Institute of Respiratory Diseases, Sir Run Run Shaw Hospital, Zhejiang University School of Medicine, Hangzhou, China; ^4^ Department of Critical Care Medicine, Sir Run Run Shaw Hospital, Zhejiang University School of Medicine, Hangzhou, China; ^5^ Department of Clinical Laboratory, The Quzhou Affiliated Hospital of Wenzhou Medical University, Quzhou People’s Hospital, Quzhou, China; ^6^ State Key Laboratory for Diagnosis and Treatment of Infectious Diseases, Collaborative Innovation Center for Diagnosis and Treatment of Infectious Diseases, The First Affiliated Hospital of Medicine School, Zhejiang University, Hangzhou, China

**Keywords:** KPC-2, hypervirulent, *K. pneumoniae*, IncP1 plasmid, *P. aeruginosa*

## Abstract

Hypervirulent *Klebsiella pneumoniae* strains are typically associated with severe infections and susceptible to most antimicrobial agents. In 2017, a carbapenem-resistant hypervirulent *K. pneumoniae* (CR-hvKP) strain was isolated from the sputum of a chronic obstructive pulmonary disease (COPD) patient in Zhejiang, China. The goal of the present study was to characterize the molecular features of the CR-hvKP isolate ZJ27003 and its *bla*_KPC-2_-harboring plasmid p27003_KPC. Antimicrobial susceptibility was evaluated using the broth microdilution and agar dilution methods. String tests, serum-killing and mouse survival assays were performed to assess virulence, and plasmid conjugation was performed by filter mating. The complete genome sequence of ZJ27003 was acquired using a hybrid assembly of Illumina and Nanopore platform data. The sequence type (ST) of this CR-hvKP isolate was identified as ST23, which exhibits hypervirulence with high serum resistance and murine infection model. The strain is also resistant to carbapenems (imipenem, meropenem and ertapenem), aztreonam and cephalosporins. Additionally, the CR-hvKP isolate carries a 36,708-bp *bla*_KPC-2_-harboring plasmid, named p27003_KPC, belonging to the P1 incompatibility (Inc) group. The backbone of p27003_KPC is similar to that of a *bla*_GES-5_-harboring *Pseudomonas aeruginosa* plasmid, in which the *bla*_GES-5_ and its surrounding regions were replaced by a *bla*_KPC-2_-containing translocatable unit derived from *Enterobacteriaceae*. The results of a conjugation assay revealed that p27003_KPC can be transferred from *K. pneumoniae* to *P. aeruginosa* PAO1 and make the recipient resistant against carbapenem. The identification of a carbapenem-resistant hypervirulent *K. pneumoniae* isolate carrying and disseminating the *bla*_KPC-2_ gene highlights a severe threat to public health.

## Introduction

*Klebsiella pneumoniae* is a major gram-negative pathogen for hospital-acquired infections in China (Kim et al., 2017). One of its unique special populations, hypervirulent *K. pneumoniae* (hvKP) is associated with severe invasive infections in healthy individuals (Shon and Russo, 2012), including pneumonia, genitourinary tract infection and septic shock (Lepuschitz et al., 2019; Walker et al., 2019). In addition, carbapenem-resistant *K. pneumoniae* (CRKP), which is associated with high morbidity and mortality, commonly limits therapeutic strategies and causes poor prognosis. HvKP strains are generally susceptible to most of antibiotic drugs and belong to K1 or K2, while ST23 *K. pneumoniae* is classified as K1 and commonly considered to be hypervirulent (Chang and Chou, 1995; Bialek-Davenet et al., 2014). In contrast, in CRKP groups, ST11 accounted for 63.9% of isolates in Zhejiang, China (Hu et al., 2020). In previous studies, hypervirulent and carbapenem-resistant phenotypes were rarely observed to co-exist in single *K. pneumoniae* strain, but recent sporadic reports of carbapenem-resistant hypervirulent *K. pneumoniae* (CR-hvKP) signaled the emergence of this new superbug. The generation of such strains is achieved through virulence or drug-resistance gene transfer *via* plasmid dissemination such that ST11 CRKP acquired a highly virulent phenotype through a virulence plasmid and *vice versa* (Yao et al., 2015; Qi et al., 2010). CR-hvKP is undoubtedly a superbug and its relative reports of which are increasing in frequency (Li et al., 2014). Thus, the occurrence and spread of CR-hvKP pose a considerable threat to public health and should be closely monitored.

*Pseudomonas aeruginosa* is another common nosocomial pathogen, and its carbapenem-resistance is intractable for clinical treatment, especially considering the intrinsic resistance of *P. aeruginosa* to many antibiotic classes. Notably, *bla*_KPC-2_-carrying plasmids have been detected in *P. aeruginosa* and can mediate carbapenem resistance (Hagemann et al., 2018; Galetti et al., 2019). Interspecies transmission of *bla*_KPC-2_-plasmids has been reported (Miriagou et al., 2003; Gao et al., 2020) in CRKP, but no studies have demonstrated that plasmids mediate *bla*_KPC-2_ gene transfer between these two notorious pathogens. In our present study, we discovered a *bla*_KPC-2_-IncP1-plasmid that can conjugate from CR-hvKP to *P. aeruginosa* and have not been reported elsewhere previously. Obviously, the *bla*_KPC-2_ plasmid will present a major problem to public health and increase the difficulty of controlling epidemics caused by carbapenem-resistant strains. Here, we described an ST23 *K. pneumoniae* isolate, carrying the IncP1 KPC-2-encoding plasmid p27003_KPC. In addition, the novel plasmid could transmit *bla*_KPC-2_ from *K. pneumoniae* to *P. aeruginosa*.

## Materials and Methods

### Patient and Isolation Data

ZJ27003 was obtained from an elderly patient with chronic obstructive pulmonary disease (COPD). This isolate was identified using an automated Vitek 2 system (BioMérieux, Marcy l’Etoile, France). This study was approved by the Ethics Committees of the Sir Run Run Shaw Hospital (20170301-3) with a waiver of informed consent because of the retrospective nature of the study.

### PCR and Sanger Sequencing

PCR and sequencing were used to screen the *bla*_KPC-2_ gene in ZJ27003 with the primers KPC-2-F (5′- ATGTCACTGTATCGCCGTCT -3′) and KPC-2-R (5′- TTTTCAGAGCCTTACTGCCC -3′).

### Antimicrobial Susceptibility Testing

The minimum inhibitory concentration (MIC) values of imipenem, meropenem, ertapenem, ciprofloxacin, tetracycline, chloramphenicol, cefepime, cefotaxime, cefoxitin, cefuroxime and gentamicin were evaluated by the broth microdilution method, and those of amikacin, aztreonam, fosfomycin, ceftazidime/avibactam, tigecycline and colistin were determined according to the agar dilution method. The guidelines of the European Committee on Antimicrobial Susceptibility Testing (http://www.eucast.org/clinical_breakpoints) and Food and Drug Administration (https://www.fda.gov) were employed to estimate colistin and tigecycline resistance, while the others were confirmed by the Clinical and Laboratory Standards Institute 2020 guidelines.

### String Test and Serum Killing Assay

The mucoviscous phenotype of this strain was estimated by stretching and evaluating a viscous string with an inoculation loop (positive, >5mm in length).

We collected fresh nonheated human serum (NHS) from 10 different healthy individuals and frozen the samples at -80°C. To ensure the accuracy of bacterial, we first calculated the cell concentration of bacterial suspension with an OD_600_ of 1, which was subsequently used for dilutions to make sure the final inocula concentration. In pre-experiments, we evaluated the bactericidal ability of the collected serum by incubating NTUH-K2044 and ATCC700603 (3×10^5^ colony forming units, CFU) into 0.3 mL of serially diluted phosphate-buffered saline (PBS)-attenuated serum solutions. Based on our pilot results, we utilized 100% NHS to carry out subsequent experiments. To ascertain ZJ27003 human serum sensitivity, the isolate was shaken until reaching mid-log phase, and samples were inoculated at 3×10^5^ CFU in 0.3mL 100% either pooled NHS or heat-inactivated human serum (HIS) for 3 h. HIS was produced through heating NHS at 56°C for 30 min. At one-hour intervals following inoculation, serially diluted bacteria were immediately plated on Mueller-Hinton agar. The serum bactericidal effect was presented as survival curves and rates, using the bacterial counts at each time point and the percentage survival values calculated when incubated with HIS were used as the denominator. In this assay, we used NTUH-K2044 as a hypervirulence control and ATCC700603 as a common strain control. This experiment was technically repeated three times.

### Murine Infection Model

The virulence of the *K. pneumoniae* strain was evaluated using BALB/c female mice weighing 20-25 g (Ziyuan, Hangzhou, China). The hypervirulence and common strain controls were the same as those used in the serum killing assay. Briefly, overnight cultures were subcultured in fresh broth at a 1:1000 dilution until reaching mid-log phase, followed by diluting the adjusted bacterial suspension (OD_600_ = 1) to 5×10^7^ with PBS. The mice were randomly placed into 3 groups, each of which had 10 mice. The grouped mice were intraperitoneally injected with 0.2 mL of prepared germ solution and observed and recorded every 12 h. The final result was visualized with GraphPad Prism. Animal protocols were approved by the Institutional Animal Ethics Committee of Zhejiang University with the number of ZJU20160154.

### Whole Genome Sequencing (WGS) and Analysis

The total DNA of ZJ27003 was extracted and then sequenced on an Illumina-HiSeqTM 2000 sequencing system (Illumina Inc, San Diego, CA, USA) based on a paired-end 2×100 bp protocol and the single-molecule real-time technique *via* nanopore platform. Unicycler was utilized to *de novo* assemble the whole genome. Gene prediction and annotation were conducted on prokka. Chromosome maps were created by CGview (https://paulstothard.github.io/cgview/), and BRIG v0.95 was used to draw plasmid comparison profiles. The whole genome was submitted on the website of Center of the Genomics Epidemiology (http://www.genomicepidemiology.org/), in order to analyze its multilocus sequence type and resistance genes. Virulence genes were detected on Pasteur (https://bigsdb.pasteur.fr). Plasmid comparison results were acquired through BLAST (http://blast.ncbi.nlm.nih.gov). Similar published ST23 CR-hvKP strain information was obtained from BacWGST (http://bacdb.org/BacWGSTdb/Search_kpn.php). The genome sequence of strain ZJ27003 has been submitted to GenBank under the accession number CP067060-CP067062.

### Conjugation

Since *P. aeruginosa* shows a high degree of natural resistance towards tigecycline, *P. aeruginosa* strain PAO1 was used as a recipient and ZJ27003 as a donor. The conjugation experiment followed the filter mating method. Mueller-Hinton agar containing tigecycline (8 mg/L) and meropenem (4 mg/L) was utilized to screen transconjugants. Transconjugant verification was performed by *bla*_KPC-2_ PCR and species identification with automated Vitek 2 system.

## Results

### Isolation of a Carbapenem-Resistant Strain

In February 2017, *K. pneumoniae* strain ZJ27003 was isolated from the sputum of an old patient who was diagnosed with COPD and hospitalized in the intensive care units of a tertiary hospital in Zhejiang, China. Drug susceptibility tests were performed and ZJ27003 showed carbapenem-resistance. The MICs was 16 mg/L for both imipenem and meropenem and reached at 32 mg/L for ertapenem (**Table 1**). Furthermore, ZJ27003 was resistant to aztreonam and cephalosporins.

**Table 1:** MICs for ZJ27003 of carbapenems and other antibiotics(mg/L).

Drug	Imipenem	Meropenem	Ertapenem	Ciprofloxacin	Tetracycline	Chloramphenicol	Cefepime	Cefotaxime	Cefoxitin
MICs	16	16	32	0.016	1	4	>32	>8	>64
**Drug**	**Cefuroxime**	**Ceftazidime**	**Gentamicin**	**C/A**	**Fosfomycin**	**Tigecycline**	**Colistin**	**Amikacin**	**Aztreonam**
MICs	>64	>32	1	2	16	0.5	<0.125	2	>64

MICs, minimum inhibitory concentrations; C/A, ceftazidime/avibactam.

### Genome Sequence Analysis

**Figure 1:**
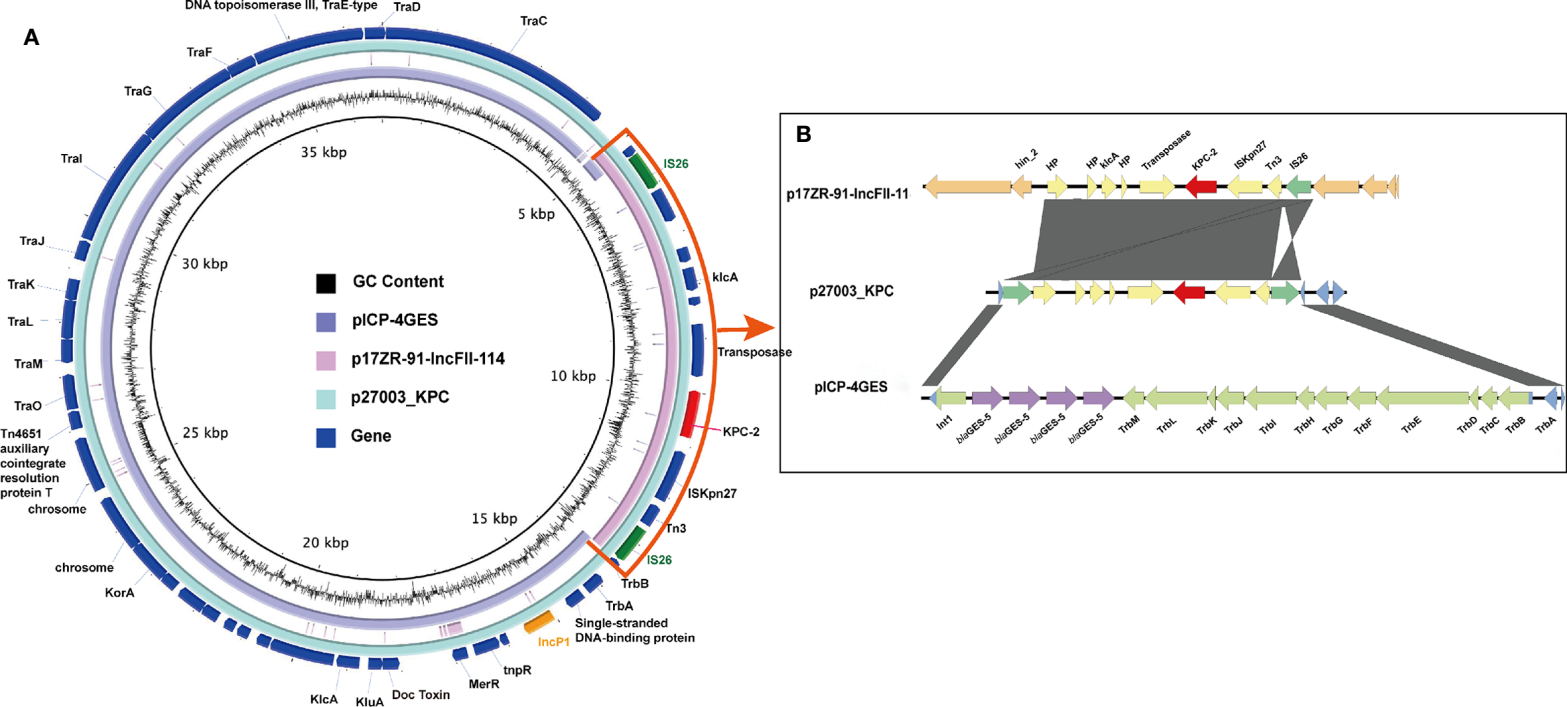
Similarity of the genetic structure of p27003_KPC with that of two other plasmids. **(A)** Alignments of similar genes on plasmids are shown as concentric parts. The outer ring shows the ORFs on p27003_KPC. *bla*_KPC-2_ is colored red, while IncP1 and IS*26* are colored orange and green, respectively. **(B)** The hybrid parts are highlighted and described in the box on the right. ORFs are illustrated as arrows. Shading areas indicate the high homology regions (≥95% identity) with the yellow or blue CDS. Orange and green boxes represent the backbone CDS on p17ZR-91-IncFII-11 and pICP-4GES, respectively.

To determine the mechanism of ZJ27003 carbapenem resistance, we performed WGS and obtained a 5,428,398-bp circular chromosome and two plasmids (141,639 bp and 36,708 bp). A total of 5,187 putative open reading frames (ORFs) and 114 RNA genes were annotated on the chromosome, while 163 and 43 putative ORFs were carried on p27003_1 and p27003_KPC, respectively. General information on the chromosome and p27003_1 is shown in **Supplementary Figure 1**. Serotyping and multilocus sequence type analyses indicated that ZJ27003 belongs to serotype K1 and ST23. In addition, among the five identified drug resistance genes, *fosA*, *bla*_SHV-190_ and *oqxAB* were located on the chromosome (**Supplementary Figure 1**) and *bla*_KPC-2_ was carried by p27003_KPC (**Figure 1**).

### An IncP1 *bla*_KPC-2_-Harboring Plasmid

p27003_KPC belongs to IncP1 and did not identify any existing plasmids on the GenBank database with high similarity and high coverage according to BLAST. Notably, a 20-kb backbone region in p27003_KPC is highly homologous to pICP-4GES (MH053445.1, comes from *P. aeruginosa*), with 99.98% identity, while the remaining components consist of a 6-kb *bla*_KPC-2_-embedded accessory region (excepted from p17ZR-91-IncFII-114, NZ_MN200129.1), which has been widely reported in *K. pneumoniae bla*_KPC-2_-plasmids.

Further exploration suggested that this integration was achieved by two IS*26* elements (two green regions in **Figure 1A**). IS*26* came from the *K. pneumoniae bla*_KPC-2_-containing translocatable unit, which duplicated itself and carried *bla*_KPC-2_ into the IncP1 plasmid. Additionally, the two IS*26* elements interrupted Int1 and TrbB, and the remaining parts could completely map the flanked sequences of *bla*_GES-5_-surrounding regions on pICP-4GES (**Figure 1B**). Overall, IS*26* helped *bla*_KPC-2_ and its accessary regions insert into the backbone of pICP-4GES and recombine a part of the antibiotic resistance gene out of the IncP1 plasmid.

### p27003_KPC Spreading Between Species

Because the backbone of p27003_KPC was similar as a *P. aeruginosa* drug-resistant plasmid, we suspect that the recombinant plasmid could bring the carbapenem resistant phenotype into *P. aeruginosa*. Plasmid conjugation was proceeded between ZJ27003 and PAO1, a carbapenem-sensitive *P. aeruginosa* strain, and the recipient was named as PAO1-pKPC. The MIC values for PAO1-pKPC changed from 1 mg/L to 8 mg/L for both imipenem and meropenem (**Table 2**). In summary, *P. aeruginosa* can change to a carbapenem-resistant strain by acquiring p27003_KPC from *K. pneumoniae*.

**Table 2:** MICs for ZJ27003, PAO1 and PAO1-pKPC of carbapenem antibiotics.

Strains/Drugs	Imipenem	Meropenem
ZJ27003	16	16
PAO1	1	1
PAO1-pKPC	8	8

PAO1-pKPC, strain PAO1 with p27003_KPC.

### CRKP ZJ27003 Virulence Genes and Virulence Assessment

**Figure 2:**
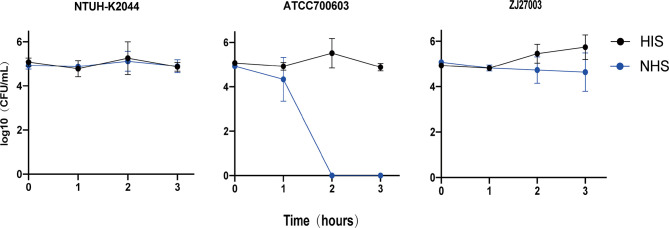
Survival of *K. pneumoniae* isolates in human serum. *K. pneumoniae* strains were incubated in 100% NHS or HIS. Samples were enumerated every 60 mins by a plate count. NTUH-K2044 was utilized as a hypervirulent strain control, while ATCC700603 was that of common strain. Triplicate independent experiments were performed for each strain.

**Figure 3:**
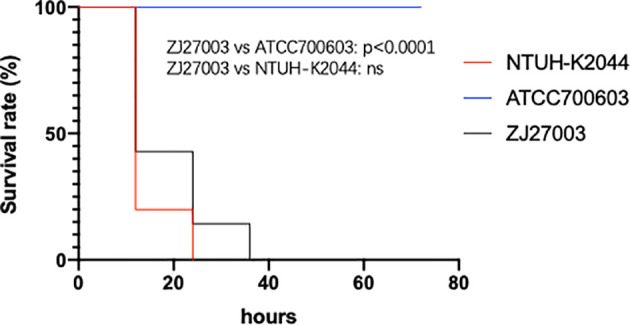
Mouse survival rates infected with *K. pneumoniae* strain ZJ27003, typical hypervirulent strain NTUH-K2044 and common strain ATCC700603, respectively. Mouse were infected intraperitoneally with 1×10^7^ CFU *K. pneumoniae* strains and monitored each 12 hours for 3 days.

Although ZJ27003 belongs to capsule type K1 and ST23, its string test is negative. Further analysis of the whole genome of ZJ27003 revealed 66 virulent-related genes. All virulence genes were harbored on the chromosome and concentrated in four large regions (**Supplementary Figure 1**). In addition, the lack of mucoid regulator gene *rmpA/rmpA2* may explain the ZJ27003 non-hypermucoviscous phenotype. Moreover, ZJ27003 lacked aerobactin, which is thought to be tightly associated with *K. pneumoniae* virulence and serum-resistance. ZJ27003 exhibited an 80% survival rate at the point of three hours (**Supplementary Figure 2**), and the survival curves (**Figure 2**) were much closer to those of the hypervirulence control (NTUH-K2044) than the common strain control (ATCC700603). Similar result was noted in murine infection model. The survival rate in mice is significantly lower than ATCC700603 while comparable to the well-known hypervirulent strain NTUH-K2044 (**Figure 3**). Similar to other ST23 *K. pneumoniae* strains, ZJ27003 displayed a high serum-resistance and mouse infection manifestation.

## Discussion

In this study, we identified an ST23 *K. pneumoniae* isolate ZJ27003 with an IncP1 *bla*_KPC-2_-harboring plasmid, p27003_KPC. The capsule type of this strain is K1 and it exhibits high human serum resistance and mouse infection manifestation. In addition, the negative string test matches the absence of the capsule regulatory gene *rmpA/A2*, which is considered interrelated with virulence (Chen et al., 2004). This absence hints that hypermucoviscosity does not always co-occur with hypervirulence in *K. pneumoniae* (Walker and Miller, 2020). In addition, all ZJ27003 virulence genes are carried on its chromosome and could be divide into the following functional categories: nutritional factors (*allABCRS* and *ybbWY*), toxins (*clbABCDEFGHIJKLMNOPQR*), iron uptake (*fyuA*, *irp1*, *irp2* and *ybtAEPQSTUX*), biofilm formation (*mrkABCDFHIJ*), ferric iron transporter (*kfuABC*) and others (metabolism-related *arc*, *fdrA*, *gcl*, *glxKR*, TIM barrel protein *hyi* and unknown *ylbEF*). Surprisingly, of the four highly virulence-related siderophore systems (yersiniabactin, aerobactin, enterobactin and salmochelin), ZJ27003 carries only yersiniabactin, which commonly exists in all *K. pneumonia*e strains and unlikely to play a role in systemic infection (Russo and Marr, 2019). In contrast, the hvKP-specific siderophores, salmochelin and aerobactin, are lacking in ZJ27003. Aerobactin is helpful for the survival of bacteria in human ascites and serum and mouse infection models (Russo et al., 2014). However, ZJ27003 exhibited high serum resistance with aerobactin absence, which implied that other genes might contribute to serum survival ability. Additional hvKP strains and further research are needed to identify those genes.

Apart from carbapenem resistance, ZJ27003 shows aztreonam and cephalosporins resistance, which could be attributed to *bla*_KPC-2_ and *bla*_SHV-190_ (Bush, 2013). In addition, for *fosA* and *oqxAB* carries limited resistance towards fosfomycin and fluoroquinolones (Wyres et al., 2020), ZJ27003 is sensitive to these two drugs.

*Klebsiella pneumoniae* carbapenemase (KPC) acts as the most prevalent carbapenemase in clinical *K. pneumoniae* isolates (Chavda et al., 2016) and could also convey carbapenem-resistant phenotype in *P. aeruginosa* (Tahmasebi et al., 2020), *Salmonella* (Miriagou et al., 2003) and other species. Its wide distribution is attributed to plasmid interspecies transmission ability. Considering that KPC seldom exists in ST23 *K. pneumoniae* (8/130 entries, BacWGST database), reports have not emerged that resistance gene carrying plasmids could conjugate among CR-hvKP and *P. aeruginosa*. Focusing on *K. pneumoniae*, the most prevalent carbapenemase-related plasmid replicon groups are IncF, A/C and X (Kopotsa et al., 2019), while IncP is rare but common in the strains from environment and exhibits a broad host range (Partridge et al., 2018). In 2015, Gang Li discovered a *bla*_KPC-2_ and *fosA* co-located IncP plasmid in a ST11 *K. pneumoniae* (Li et al., 2015), and IncP plasmid could also be duplicated in *P. aeruginosa* (Dai et al., 2016). In other words, IncP group plasmids can be replicated, stabled and transcribed in both *K. pneumoniae* and *P. aeruginosa*. This group of plasmids might have the capacity to transfer and exchange between *K. pneumoniae* and *P. aeruginosa.* With this conjecture, we excluded the *bla*_KPC-2_-containing translocatable unit from p27003_KPC, and the coverage and identity were increased at 98% and 100% respectively, which hinted that the new plasmid might be a hybrid from pICP-4GES (a *P. aeruginosa* plasmid) and *K. pneumoniae bla*_KPC-2_-containing translocatable region. Conjugation experiments proved a part of our conjecture that the IncP1 plasmid could transfer from *K. pneumoniae* to *P. aeruginosa*.

According to the NCBI database, we found that the backbone of p27003_KPC is also highly similar to that of pDCT28, which is isolated from ocean sediment (Hayes et al., 2016). Additionally, this plasmid backbone exists in *P. aeruginosa*, *Achromobacter xylosoxidans* (KJ588780.1), *Variovorax paradoxus* (Zhang et al., 2016) and *Burkholderia ambifaria* (Johnson et al., 2015). We further conjugate p27003_KPC into *P. aeruginosa* PAO1, and our experiment reveal that p27003_KPC can duplicate and multiply in *P. aeruginosa* and make recipients carbapenem-resistant. Based on this finding, we suspect p27003_KPC might directly bring *bla*_KPC-2_ into other species directly, which will lead to wider and easier dissemination of *bla*_KPC-2_.

## Conclusion

In conclusion, we isolated the CR-hvKP strain ZJ27003 from an 85-year-old male. ZJ27003 is an ST23 K1 non-hypermucoviscous *K. pneumoniae* and exhibits high serum resistance and mouse infection manifestation. In addition, the carbapenem-resistant phenotype of this strain is mediated by the novel IncP1 *bla*_KPC-2_ plasmid p27003_KPC. This plasmid can conjugate from *K. pneumoniae* to *P. aeruginosa*. Notably, CR-hvKP carrying and disseminating *bla*_KPC-2_-harboring plasmids will pose a big challenge to public health.

## Data Availability Statement

The datasets presented in this study can be found in online repositories. The names of the repository/repositories and accession number(s) can be found below: NCBI Genbank; CP067060-CP067062.

## Ethics Statement

This study was approved by the 79 Ethics Committees of the Sir Run Run Shaw Hospital (20170301-3) with a waiver of informed consent because of the retrospective nature of the study.

## Author Contributions

YY, YJ, and JZ isolated and provided strain ZJ27003 and designed the study and experiments. RY and YL carried out the assays. YZ and PL analyzed the data. RY and SJ drafted and revised the manuscript. All authors contributed to the article and approved the submitted version.

## Funding

This work was supported by the National Natural Science Foundation of China (grant No. 81672067, 81830069, and 81902102), the National Science and Technology major project (2018ZX10714002) and Zhejiang Province Medical and Health project (Grant No. 2019ZD023).

## Conflict of Interest

The authors declare that the research was conducted in the absence of any commercial or financial relationships that could be construed as a potential conflict of interest.
