# Supplementary figures and images for: A Sequence Type 23 Hypervirulent Klebsiella pneumoniae Strain Presenting Carbapenem Resistance by Acquiring an IncP1 bla KPC-2 Plasmid
Source: Front Cell Infect Microbiol. 2021 Jun 1;11:641830. doi: 10.3389/fcimb.2021.641830 (PMC8204043; doi:10.3389/fcimb.2021.641830)

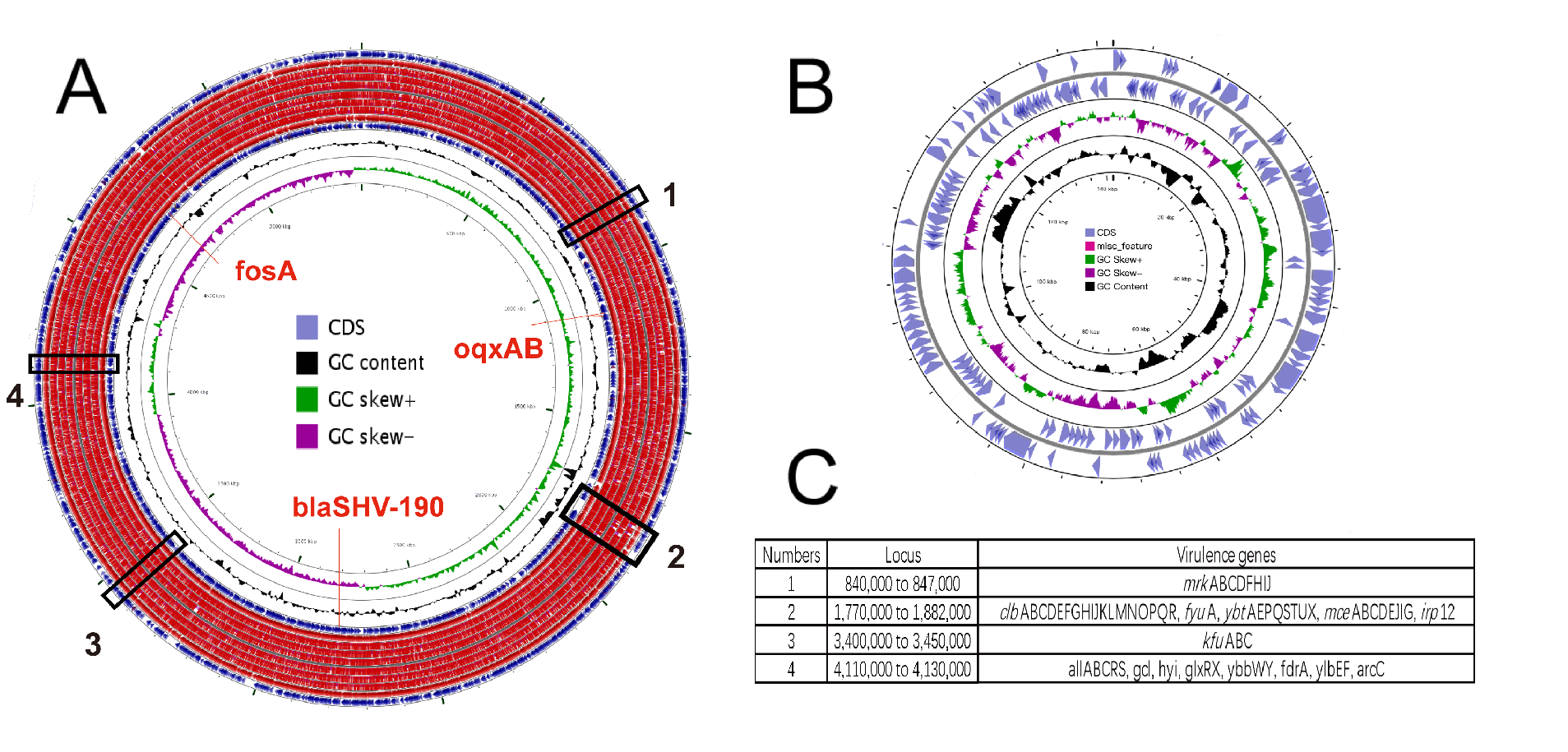

Supplement: Supplementary file 1 [file Image_1.tif]

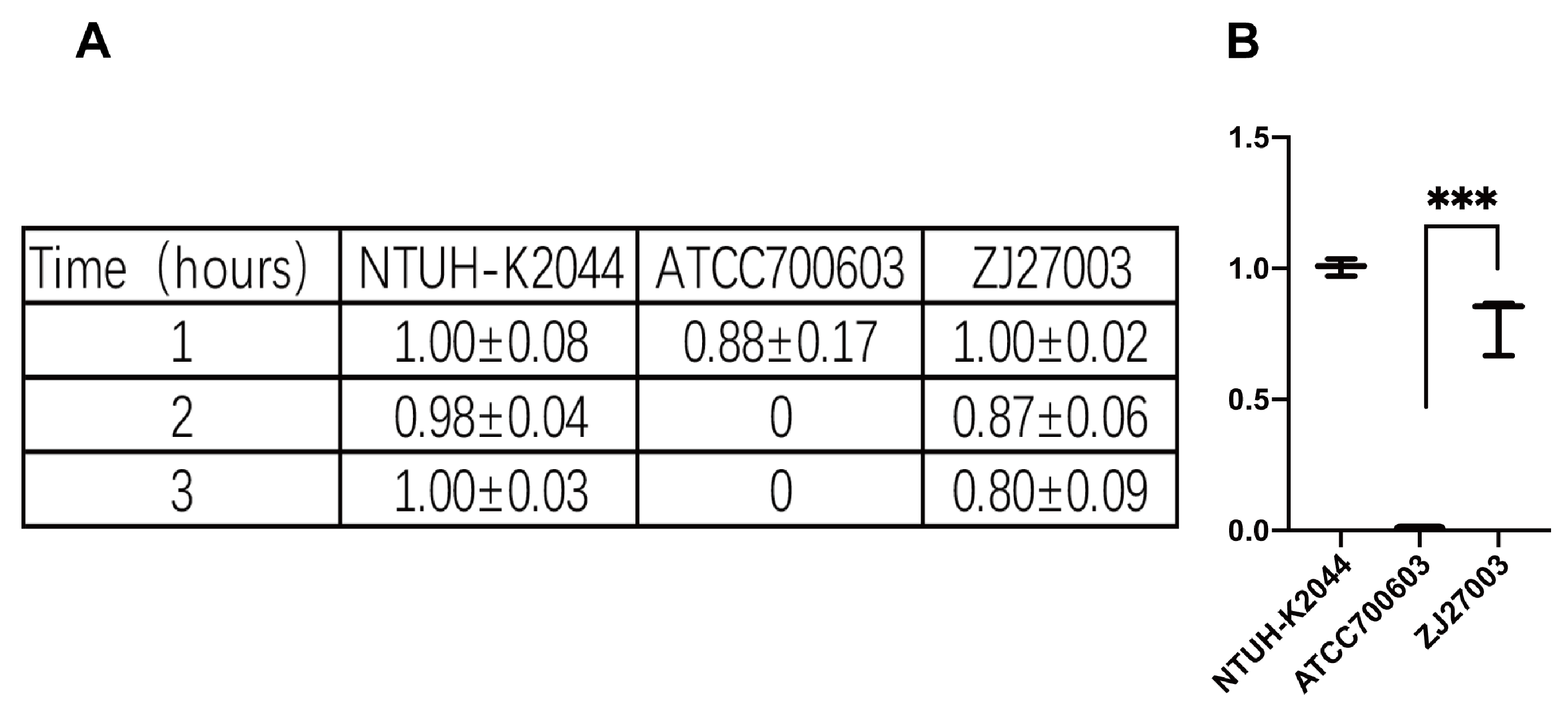

Supplement: Supplementary file 2 [file Image_2.tif]
