# Supplementary material for: A Sequence Type 23 Hypervirulent Klebsiella pneumoniae Strain Presenting Carbapenem Resistance by Acquiring an IncP1 bla KPC-2 Plasmid
Source: Front Cell Infect Microbiol. 2021 Jun 1;11:641830. doi: 10.3389/fcimb.2021.641830 (PMC8204043; doi:10.3389/fcimb.2021.641830)
Supplement: Supplementary file 3 [file Table_1.docx]

**Supplementary Figure 1.** Map of the ZJ27003 chromosome (A) and 141,639-bp plasmid p27003_1 (B). The three orange labels in the left circle (A) indicate resistance genes carried on the chromosome. The four black boxes on the left circle locate virulence genes on the chromosome, and the four numbers correspond to four genetic structures detailed in the right table (C).

**Supplementary Figure 2.** Survival rates of *K. pneumoniae* strains in serum. (A) Serum resistance of the three isolates at each time point. Percent survival was calculated following each hour of incubation in 100% NHS, and normalized by values obtained from inoculation with HIS as 100%. (B) Visualize survival rates for each isolate at 3 h. The data were obtained from triplicate assays for each isolate. Bars represent the SE of the mean. **P* < 0.01.
